# Planned vaginal birth with epidural analgesia in obese women: Effects on maternal and fetal outcomes. A retrospective cohort study

**DOI:** 10.1016/j.eurox.2026.100466

**Published:** 2026-06-04

**Authors:** Ewout C. van der Wal, Johannes J. Duvekot, llse J.J. Dons-Sinke, Robert J. Stolker, Sanne E. Hoeks, Caroline D. van der Marel

**Affiliations:** a Department of Anesthesiology, Erasmus MC, Rotterdam, the Netherlands; b Department of Obstetrics and Gynecology, Division of Obstetrics and Prenatal Medicine, Erasmus MC, Rotterdam, the Netherlands

**Keywords:** Obesity, Vaginal delivery, Epidural analgesia, Pain, Labour

## Abstract

**Background:**

Obese pregnant women (body mass index ≥ 30 kg/m^2^) often receive epidural analgesia (EA) during their deliveries. However, there is scarce evidence on the effect of EA on maternal and perinatal outcomes in these women. The goal of this study is to evaluate outcomes in obese women receiving EA during their planned vaginal deliveries.

**Methods:**

Patient records of obese women undergoing a planned vaginal delivery from January 2018 until December 2023 in a tertiary care center were screened. Women were stratified into two groups depending on whether or not they received EA during their deliveries. EA was provided after shared decision making between provider and mother. Baseline characteristics and perinatal outcomes were collected for both groups. Primary outcomes were conversion to cesarean section (CS) and reduced Apgar scores (<7 at 5 min). Secondary outcomes were mode of delivery, type of anesthesia, estimated blood loss, episiotomies, perineal ruptures, hospital length of stay, fetal umbilical artery pH and base excess. Using inverse probability of treatment weights, primary outcomes per group were adjusted for baseline characteristics in a logistic regression model.

**Results:**

259 women received EA, 144 did not. Women with EA had lower gravidity and parity, more often induction of labor and delivered children with higher birthweights. In total 88 (22%) women in both groups required secondary CS. General anesthesia was avoided for all 64 women with EA. In the non-EA group, general anesthesia was used for seven out of 24 women. After adjustment, conversion to CS (OR 0.984, CI 0.542–1.786, P = 0.957) and reduced Apgar scores (OR 1.138, CI 0.375–3.458, P = 0.820) were not significantly different. Secondary outcomes (unadjusted) showed several differences. EA was associated with more vacuum deliveries (16% vs 2%, P < 0.001), more episiotomies (18% vs 7%, P = 0.003), higher mean blood loss (523 ml vs 351 ml, P < 0.001), and longer mean length of hospital admittance (4.3 days vs 3.5, P < 0.001).

**Conclusions:**

Women with a BMI ≥ 30 kg/m^2^ receiving EA for planned vaginal delivery showed similar conversion to CS and similar Apgar scores, when compared to obese women without EA. Secondary maternal outcomes were less beneficial for the group receiving EA, but fetal outcomes were not. When conversion to surgical delivery was required, general anesthesia was avoided in 100% of women with EA, while 29% of women without EA required general anesthesia, although fetal outcomes were not worse in this group. In conclusion, early epidural placement for intended vaginal delivery in obese women is safe for mother and child and may prevent the need for general anesthesia for conversion to CS.

## Highlights

• Planned vaginal delivery in obese women has similar conversion to cesarean section with and without epidural analgesia.
• Maternal outcomes may be worse when receiving epidural analgesia, but fetal outcomes are similar.
• Early epidural placement may prevent the need for general anesthesia for conversion to cesarean section in emergencies.

## Introduction

Obesity (defined as a body mass index ≥ 30 kg/m^2^), is increasing world-wide. [1] Obstetric health care providers are aware of this phenomenon, as obesity increases the risk of many complications and worsens outcomes during and after pregnancy. [2], [3], [4], [5] Anesthesiologists have to take obesity into account when providing intra-partum anesthesia. A major problem is the increased chance of cesarean section (CS) with increasing body mass index (BMI), and thus an increased need for surgical anesthesia. [4], [6], [7], [8]

However, anesthesia has its own unique problems in obese pregnant women. [9], [10], [11], [12], [13] General anesthesia is overall not recommended for delivery through CS because obesity and pregnancy are both associated with difficulties in airway management. [8], [14], [15] In order to avoid general anesthesia, neuraxial anesthesia is a good alternative for conducting CS. Spinal or combined spinal-epidural (CSE) anesthesia are two possible modalities. In obese women performing spinal anesthesia is often more challenging and might take extra time. The time is not always available when urgent delivery is needed, and thus general anesthesia will still be needed. Therefore, timely administration of epidural analgesia (EA) during labor is advised by several guidelines in the field of obstetric anesthesia. [16], [17], [18] An epidural catheter correctly placed during planned vaginal delivery may be used when conversion to surgical delivery is needed, in lieu of general anesthesia.

EA overall is deemed safe but has several known side-effects and risks. [19] In obesity specifically, epidural insertion can be more difficult and yields a higher risk at post-epidural hypotension. [20], [21], [22] Aside from these known side-effects, the effect of labor EA on mother and child is studied in the general population but not specifically in obese women. Nonetheless, EA for child delivery is recommended by guidelines in this population. [23] The primary goal of these recommendations, apart from pain relief, is creation of safe (anesthetic) conditions, anticipating the event that (acute) surgical delivery is needed. However, the guidelines mainly rely on expert opinion. Literature with evidence based conclusions on definite outcomes for mother and offspring is scarce. [16] Our recent systematic review concluded that there is no definite answer to this issue. [24] Furthermore, it is important to practice evidence-based medicine and to inform obese women on all specific relevant risk and benefits, especially since placement of an epidural catheter is an invasive measure. Therefore, specific evidence for this population is needed.

This retrospective cohort study evaluates the maternal and perinatal outcomes of EA during planned vaginal delivery in a population of obese women.

## Methods

We followed the Strengthening the Reporting of Observational Studies in Epidemiology (STROBE) statement guidelines for reporting observational studies. [25] This study was approved by the Institutional Review Board of the Erasmus MC (MEC−2020–0983). We obtained all information retrospectively and no patient interventions were done, therefore informed consent was not needed.

**Data collection** Our study was performed in the Erasmus University Medical Center, the largest perinatal center in the Netherlands with a specialized children’s hospital. It is also a tertiary and quaternary referral center.

The incidence of EA in the Netherlands is relatively low (23% of all deliveries) compared to other high-income countries. [26] Therefore, Dutch hospitals are suitable for cohort studies on this matter, since there are sufficient women without EA to form a control group.

The birth records from January 2018 until December 2023 were screened for women that had high BMI as one of their medical indications for in-hospital delivery. Subsequently, these data were combined with individual patient files. We included women if their BMI was ≥ 30 kg/m^2^. Exclusion criteria were delivery through elective CS, twin or higher order pregnancy, intrauterine fetal demise or a non-intervention policy, fetal gestational age < 24 weeks, multiple congenital abnormalities, and maternal age under eighteen.

We retrieved the following patient information: age, gravidity, parity, gestational age, birthweight, estimated blood loss, induction of labor, method and timing of analgesia or anesthesia, type of delivery), occurrence of episiotomy or rupture of the perineum, and duration of hospital stay. In the case of CS or vacuum delivery, indication was also extracted and divided into either maternal indication (arrested labor, pre-eclampsia, infection, fatigue) or fetal indication (fetal distress). Age, height, and BMI at the time of the first intake at the hospital were also extracted (during the start of pregnancy). Regarding the newborn, we retrieved Apgar scores at one and five minutes, birthweight, umbilical artery blood pH and base excess (BE). Following retrieval, we anonymized all patient data.

**Patient interventions** In the Netherlands, all women with a BMI above 35 are indicated to deliver in hospital. Specifically in our hospital, high-risk women including morbidly obese women (BMI ≥ 40 kg/m^2^) and obese women with a BMI ≥ 35 kg/m^2^ and comorbidity, are routinely seen by an obstetric anesthesiologist during their pregnancies. During this visit the women are thoroughly screened and different methods of analgesia are discussed, including the risks and benefits. For obese women, EA is generally recommended by the anesthesiologist (as per local guideline), however the final choice regarding (mode of) analgesia is at the woman’s own discretion. Even though remifentanil is relatively contra-indicated in obese women, it is still provided when specifically asked by the woman.

In our center, with approximately 1800 deliveries per year, labor analgesia and deliveries in general are under the responsibility of a specialized team with dedicated obstetric anesthesiologists, gynecologist-perinatologists, labor nurses, and midwives with a 24/7 coverage. After start of labor, when requested by the patient, EA is provided by the anesthesiologist using a standardized protocol. Insertion is done using a 18-gauge Tuohy needle using sterile technique around level L3-L4. After successful placement and negative aspiration, a test dose of 3 ml 1% lidocaine is administered. This is followed by a 10 ml dose of 0,1% ropivacaine with 1,0 µg sufentanil/ml after five minutes, only if the initial test dose does not already provide a change in sensory block. The total dose is increased until the block level is sufficient. The patient is then provided with a patient controlled epidural analgesia (PCEA) pump with medication and settings as shown in Table 1. Block height is routinely tested after epidural insertion. Whenever EA fails, the catheter is either repositioned or replaced with a new catheter until analgesia is sufficient.

**Table 1:** Analgesia methods and dosing.

Vaginal delivery						
Pethidine		**Remifentanil**			**Epidural anesthesia**	
Intramuscular	• Dosing as indicated by physician, usually 50–100 mg.	Intravenous (IV), patient controlled analgesia.	• Medication: 20 mcg/ml remifentanil • Bolus: 30 µg over 30 s • Lockout time: 3 min		Epidural, patient controlled epidural analgesia.	• Medication: 0.1% ropivacaine + 1 µg/ml sufentanil • Continuous: 5 ml/h • Bolus: 5 ml • Lockout time: 30 min
Secondary C-section						
Spinal anesthesia				**Epidural anesthesia (pre-existing catheter)**		
• 4 ml 0.5% bupivacaine heavy + 1 ml 5 µg/ml sufentanil • 1.6–2.5 ml				• 0.8% ropivacaine + 1 µg/ml sufentanil • 10–20 ml		
Emergency secondary C-section						
General anesthesia, rapid sequence induction				**Epidural anesthesia (pre-existing catheter)**		
• Thiopental 5 mg/kg IV • Succinylcholine 1 mg/kg IV • IV opioids after childbirth				• 2% lidocaine • 10–20 ml		

For women requiring an emergency CS, neuraxial anesthesia is the preferred modality. In women with a pre-existent epidural catheter, an epidural bolus of LA is given as shown in Table 1. Women without an epidural catheter receive either spinal anesthesia, CSE, or general anesthesia with a rapid sequence induction (Table 1). After providing neuraxial anesthesia, effectivity and block height is always tested with a cold element using a standardized method.

Primary outcomes were incidence of conversion to CS, and lowered Apgar scores at minute five. Apgar scores < 7 was considered reduced. We chose Apgar score since it’s a clinically relevant parameter, widely used, and data was very complete for this outcome. Furthermore, umbilical pH may even be normal for neonates with a bad start.[27] Secondary outcomes were mode of delivery and type of anesthesia, estimated blood loss, episiotomy, perineal rupture, hospital length of stay, fetal umbilical artery pH and BE.

**Statistical methods** Statistical analysis was done in SPSS. [28] Included women were stratified into two groups: women receiving EA and women without EA. Baseline characteristics were calculated per group and tested for difference. Standardized mean differences (SMD) were calculated a first time without adjustment, and again after adjustment. The SMD threshold was < 0.1. We tested normally distributed and non-normally distributed nominal outcome data with respectively an independent samples T-test and a Mann-Whitney *U* test. Categorical data were tested using a Chi-squared test.

A propensity score was calculated for prediction of incidence of EA per case. [29] We used all available baseline characteristics except birthweight for propensity score determination. Due to the small population this study, propensity score was not used for matching. Instead, the score was used to generate inverse probability of treatment weights (IPTW). [30] This weighting was used in a binary logistic model with a Wald Chi-Square test for significance. With this model, odds ratios were calculated to ascertain the likelihood of influence caused by EA on the primary outcomes. P values of < 0.05 were considered statistically significant.

## Results

From January 2018 until December 2023, 588 pregnant women with a BMI ≥ 30 kg/m^2^ were identified. 403 of these women intended to give birth vaginally and met our inclusion criteria. EA was used in 259 (64%) women. 144 (36%) women did not receive EA, of which ten received opioid analgesia. All women requesting EA received a sufficiently working catheter. 69 (27%) of the women receiving EA had primary/early catheter placement (<3 cm cervical dilatation).

Baseline characteristics of included women are seen in Table 2. Only length had a significant SMD (<0.01), all other baseline characteristics were not comparable between the EA and non-EA groups. After IPTW-adjustment, all baseline characteristics were comparable except birthweight (which was not used for correction) and parity, implicating a well-adjusted model.

**Table 2:** Baseline characteristics.

	**Unweighted**				**Weighted**		
**Baseline characteristics**	**Epidural analgesia (n = 259)**	**No epidural analgesia (n = 144)**	**SMD**		**Epidural analgesia**	**No epidural analgesia**	**SMD**
	*mean±SD / %*	*mean±SD / %*			*mean±SD / %*	*mean±SD / %*	
**Age**	31 ± 5	32 ± 5	0.212		32 ± 5	31 ± 5	**0.041**
**Weight (kg)**	113 ± 24	108 ± 22	0.215		112 ± 23	111 ± 23	**0.024**
**Length (cm)**	166 ± 7	166 ± 6	**0.093**		166 ± 7	166 ± 6	**0.015**
**BMI (kg/m2)**	40.9 ± 7.5	39.5 ± 7.6	0.184		40.5 ± 6.8	40.5 ± 7.9	**0.001**
**Gestational age at birth**	39.0 ± 1.6	38.0 ± 3.3	0.364		38.8 ± 1.7	38.6 ± 2.6	**0.074**
**Gravidity**	2.6 ± 1.9	3.5 ± 2.0	0.450		3.2 ± 2.4	3.0 ± 1.9	**0.085**
**Parity**	0.9 ± 1.2	1.7 ± 1.5	0.565		1.4 ± 1.8	1.25 ± 1.3	0.103
**Induction of labor**	185 (71%)	72 (50%)	0.450		62%	64%	**0.040**
**Birthweight (g)**	3422 ± 546	3154 ± 770	0.403		3362 ± 580	3269 ± 671	0.149

**Fig. 1:**
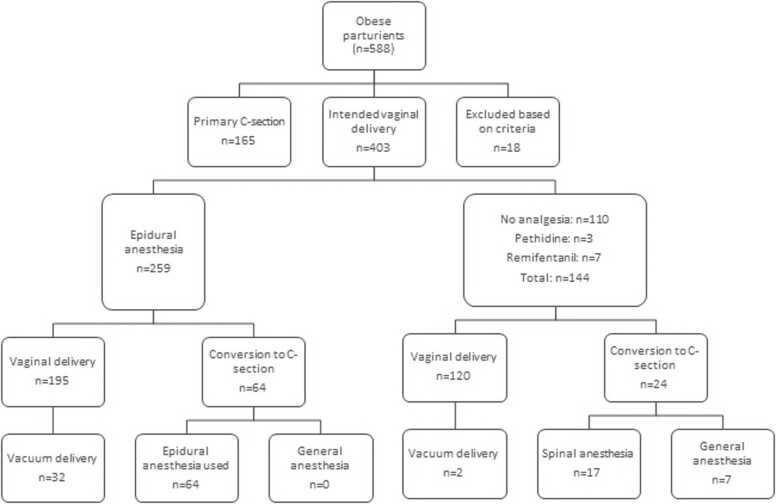
Inclusion and delivery flowchart.

Conversion to CS was required for 88 (22%) of all included women, of which 49 cases (56%) were on maternal indication and 39 (44%) due to fetal distress. In all instances where secondary CS was needed and the woman had already received an epidural catheter (n = 64), the catheter was used successfully for surgical anesthesia. In the group of women without an epidural catheter requiring conversion (n = 24), seven (29%) received general anesthesia and seventeen (71%) received spinal anesthesia. A detailed flowchart of patient inclusion, modes of anesthesia, and modes of delivery is demonstrated in Fig. 1.

Unadjusted outcomes are shown in Table 3. For some women, not all information regarding outcomes was retrievable. CS was not significantly higher in each group. However, as seen in Table 3, in the EA group there were significantly more vacuum deliveries and episiotomies. 32 out of 34 (94%) vacuum deliveries were due to fetal distress. Blood loss and length of hospital stay were also higher in parturients receiving EA (Table 3). Regarding fetal outcomes when comparing EA to non-EA, mean Apgar scores (9.4 ± 1.1 vs 9.2 ± 1.3, P = 0.203) and incidence of an Apgar score below seven (4.9% vs 3.5%, P = 0.495) did not differ significantly, however children in the EA group had a significantly lower umbilical artery pH and BE (Table 3).

**Table 3:** Outcomes.

**Outcome**	**Epidural analgesia**	**No epidural analgesia**	**P**
Surgical delivery (n = 403)	64 (25%)	24 (17%)	0.061
Vacuum delivery (vaginal group, n = 315)	32 (16%)	2 (2%)	**< 0.001**
Vacuum or surgical delivery (n = 403)	96 (37%)	26 (18%)	**< 0.001**
Perineal rupture or episiotomy (n = 403)	114 (44%)	51 (36%)	0.091
Perineal rupture (n = 400)	69 (27%)	41 (29%)	0.696
Episiotomy (n = 400)	45 (18%)	10 (7%)	**0.003**
Blood loss (ml)	523 ± 481 (n = 258)	351 ± 356 (n = 143)	**< 0.001**
Length of hospital stay (days)	4.3 ± 3.7 (n = 259)	3.5 ± 5.2 (n = 144)	**< 0.001**
Apgar score minute 1	8.2 ± 1.6 (n = 254)	8.1 ± 1.9 (n = 142)	0.892
Apgar score minute 5	9.4 ± 1.1 (n = 256)	9.2 ± 1.3 (n = 143)	0.203
Umbilical artery pH	7.22 ± 0.89 (n = 246)	7.26 ± 0.09 (n = 133)	**< 0.001**
Umbilical artery BE	−5.1 ± 3.7 (n = 243)	−4.3 ± 3.0 (n = 128)	**0.016**

After adjustment based on the inverse probability of treatment weights, women receiving EA were not significantly more likely to receive a CS (odds 0.966, CI 0.537–1.738). The second outcome showed odds 0.883, CI 0.289–2.697) for a lowered Apgar score at minute 5 in children from women with EA.

In the non-EA group, seven women received general anesthesia for conversion to CS. In only one of these cases neuraxial anesthesia was contra-indicated due to several patient-related factors, and general anesthesia was required. In all six other cases extreme urgency was required due to fetal distress, therefore spinal anesthesia was not attempted. In none of these cases maternal airway problems were experienced.

## Discussion

The objective of this study was to evaluate the effect of EA on maternal and perinatal outcomes in a population of obese parturients.

Our comparison included 403 women total. 259 (64%) of these women received EA. This percentage was higher than in the total (obese and non-obese) population, where EA was used in ±40% of all women. The EA group had several statistically significant different baseline characteristics. Primary outcomes before and after adjustment for baseline characteristics were similar, but secondary outcomes were less beneficial in the EA group.

The total number of included women (403) was relatively low compared to the total number of in-hospital deliveries (±10.000) in this period. This can be explained due to the inclusion process where only women were included if high-BMI was scored as a medical indication (or one of the medical indications) for clinical delivery. Therefore, we might have missed women who had a BMI between 30 and 35.

Most differences in baseline characteristics between both groups were expected. Women receiving EA were lower in gravidity and parity and more often were induced, which can be explained by the fact that these women generally have a longer duration of labor compared to women with more previous deliveries. Longer labor duration increases the chance of a maternal request for analgesia. Higher fetal birthweight is also associated with increased use of EA. Another explanation is that obstetric providers prefer to provide women with epidural anesthesia if they have any predictive factors for complicated labor. Age, weight, length, BMI, and gestational age at birth were similar between groups.

Findings regarding baseline characteristics might be an indication that maternal request for EA may be predicted using clinical variables. Providers might take this into account when providing patient education. Women that are likely to request EA, should receive sufficient information beforehand.

In the EA group, 25% required conversion to CS, while 17% in the no EA group required CS. This difference was not statistically significant (adjusted odds 0.966, CI 0.537–1.738). However, in the epidural group, all women received anesthesia through their already inserted epidural catheter. In the group without EA, emergency CS (under general anesthesia) was performed in seven out of 24 cases (29%). This shows that the use of EA may prevent the need for general anesthesia (with all its associated risks) and confirms findings by one previous study. [31] When looking at a general population of parturients, a large 2018 Cochrane analysis also reports no increase in incidence of CS with or without EA. [19] Interestingly, our cohort shows no decreased fetal outcomes after delivery under general anesthesia as compared to neuraxial anesthesia. We believe this may be explained due to our institutions specialized obstetrical teams using strict protocols, well-timed decision making, and the use of short-acting anesthetics.

Moreover, there is no reason to believe EA has a negative influence on the fetus, as the incidence of a low Apgar scores was not significantly increased in the EA group (odds 0.883, CI 0.289–2.697). Only one other recent study looks at this outcome for obese women with and without EA, and also reports similar Apgar scores in both groups. [31] That study compares early and late EA to no EA, and is the only known recent study looking at influence of labor EA in obese women. Therefore, we think the influence of EA in obese women may be comparable to that in the general population of women giving birth.

Secondary maternal outcomes were generally less beneficial in the EA group. There were significantly more vacuum deliveries and episiotomies, possibly due to the higher birth weight in this group. At our institution, an episiotomy is routinely performed when vacuum delivery is needed, therefore these two outcomes are closely related. Another related outcome is the higher mean blood loss, which might be explained by the higher number of episiotomies. Length of hospital stay was also higher for women receiving EA. All these differences may be explained by differences in demographics per group. The number of vacuum deliveries in the EA group (16%) was also higher when compared to the incidence in general the low-risk population in the Netherlands (8.2%), but can be explained by the general high-risk population in this study, and higher incidence of vacuum delivery is a known risk for obese women. [32] Vacuum delivery may also prevent conversion to CS which is preferably avoided, especially in obese women.

Regarding secondary fetal outcomes, only umbilical artery pH and BE were significantly lower in the epidural group, but we do not consider this finding clinically significant as only umbilical artery pH < 7 is considered to be a predictor of worse fetal outcome. [33] Moreover, mean Apgar scores at minute one and five are lower in the no-epidural group, but this finding is not significant. This also shows that EA does not seem to result in worse neonatal outcomes.

There was no loss of follow-up, however for some women not all baseline characteristics and outcome information were retrievable. Due to the design of this study and left-out inclusions, selection bias is possible but reduced by using adjustment of the primary outcome. Baseline characteristics that were not retrieved and other (unmeasurable) factors not incorporated into the propensity might have influenced our reported outcomes. Several missing baseline characteristics and outcomes were the stage of labor and cervical dilation at admission, length of labor stages, indications for induction of labor, and the number of epidurals requiring repositioning/replacement. Administration of EA was chosen using shared decision-making between women and providers. This is a major limitation of any retrospective study on this subject. Women that are more likely to need assisted or surgical delivery based on clinical characteristics, will be advised to receive EA by their providers.

All included women gave birth in our hospital, which is a tertiary and quaternary academic center in the Netherlands. This hospital is one of several reference centers in this region for obese women. However, many of these women had comorbidities other than obesity, possibly resulting in some inclusion bias. This might also explain the high induction rates in both groups (71% with EA and 50% without).

## Conclusion

Women with a BMI ≥ 30 kg/m^2^ receiving EA for planned vaginal delivery showed similar conversion to CS and similar Apgar scores, when compared to obese women without EA. Secondary maternal outcomes were less beneficial for the group receiving EA, but fetal outcomes were not. When conversion to surgical delivery was required, general anesthesia was avoided in 100% of women with EA, while 29% of women without EA required general anesthesia. However, fetal outcomes are not worse in this group. In conclusion, early epidural placement for intended vaginal delivery in obese women is safe for mother and child and may prevent the need for general anesthesia for conversion to CS.

## CRediT authorship contribution statement

**Dons-Sinke Ilse J.J.:** Writing – review & editing, Conceptualization. **Robert J. Stolker:** Writing – review & editing, Supervision, Conceptualization. **van der Wal Ewout C.:** Writing – original draft, Methodology, Formal analysis, Conceptualization. **Duvekot Johannes J:** Writing – review & editing, Supervision, Conceptualization. **Sanne E. Hoeks:** Writing – review & editing, Methodology, Formal analysis, Data curation. **Caroline D. van der Marel:** Writing – original draft, Resources, Project administration, Conceptualization.

## Declaration of Competing Interest

The authors declare that they have no known competing financial interests or personal relationships that could have appeared to influence the work reported in this paper.
